# Supplementary material for: Degraded myelin is associated with cold hypersensitivity in paclitaxel-induced peripheral neuropathy
Source: bioRxiv. 2026 Jun 2:2026.05.29.728780. Preprint. [Version 1] doi: 10.64898/2026.05.29.728780 (PMC13252001; doi:10.64898/2026.05.29.728780)
Supplement: Supplement 1 [file media-1.pdf]

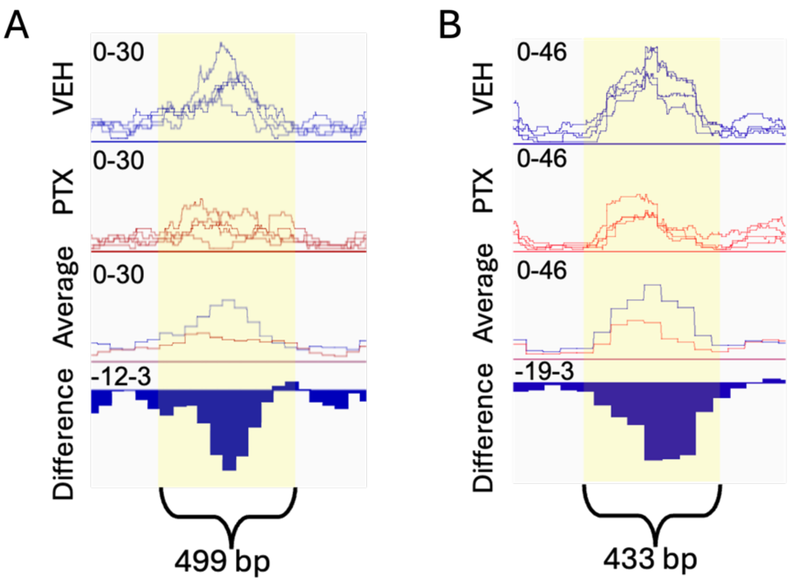

Supplemental Figure 1 Differential chromatin accessibility at regions of interest during persistent cold hypersensitivity. Differentially chromatin accessibility at **(A)** Chr3:87986750-87987249 and **(B)** Chr8:94703923-94704356. (n=4/group).

Region of interest	Forward primer	Reverse primer
Chr3:87986750-87987249	TGGAAGATCTTTGTTCTTGTCGCCTGGCTTTT	GCATACGCGTCCTGAGAGTGCCTCCGTTCC
Chr8:94703923-94704356	TGGAAGATCTCCCCGCTCCTGGAATTCTTTTT	GCATACGCGTGGACCTGATTGCCACGTACC

Supplemental Table 1 Primers for cloning. Primers used for restriction enzyme cloning of regions Chr3:87986750-87987249 and Chr8:94703923-94704356.

Gene Name	Entrez ID	log2FC of transcript	p value of transcript	Region	log2FC of region	p value of region	Region width	Annotation
5730507C01Rik	236366	1.2346	0.001534	chr12:18695785-18696079	0.2205	0.005152	295	Intergenic
Agrn	11603	-0.3271	0.001364	chr4:156197062-156198249	-0.2492	0.005931	1188	promoter-TSS
Bach1	12013	-0.3042	0.026710	chr16:87801299-87801468	-0.2227	0.002134	170	Intergenic
Bcan	12032	-0.2476	0.039889	chr3:87986750-87987249	-0.2693	0.003349	500	TTS
Bcan	12032	-0.2476	0.039889	chr3:87994224-87994763	-0.2215	0.006327	540	intron
Chdh	218865	-0.4947	0.000009	chr14:30133107-30133655	-0.2222	0.007840	549	intron
Cldn19	242653	-0.5809	0.000144	chr4:119247507-119247928	-0.2458	0.006303	422	intron
Clic4	29876	-0.3078	0.005280	chr4:135272242-135273236	-0.2798	0.001167	995	promoter-TSS
Cotl1	72042	-0.2946	0.048772	chr8:119840095-119841087	-0.2516	0.006363	993	promoter-TSS
Csmd2	329942	-0.3096	0.011208	chr4:128087134-128087558	-0.2523	0.003629	425	intron
Ddit4	74747	-0.4405	0.002456	chr10:59943527-59944016	-0.2450	0.003928	490	Intergenic
Elovl1	54325	-0.2919	0.021459	chr4:118427734-118428603	-0.2999	0.001040	870	promoter-TSS
Gprc5b	64297	-0.3562	0.004889	chr7:119023648-119023876	-0.2063	0.008901	229	Intergenic
Iqsec1	232227	-0.3093	0.048573	chr6:90716058-90716741	-0.2364	0.008779	684	5' UTR
Itga6	16403	-0.3436	0.000569	chr2:71786576-71787918	-0.3115	0.001180	1343	exon
Itgav	16410	-0.2843	0.005529	chr2:83754046-83754544	-0.2561	0.002580	499	intron
Kcnc3	16504	-0.5548	0.000391	chr7:44590512-44591432	-0.3083	0.000848	921	exon
Kcnip4	80334	0.2432	0.024940	chr5:49583707-49583971	0.1868	0.009900	265	Intergenic
Mbp	17196	-0.3928	0.012020	chr18:82525917-82526487	-0.2307	0.008557	571	intron
Mug1	17836	2.3526	0.025404	chr6:121780681-121780973	0.1848	0.009458	293	Intergenic
Papln	170721	-0.8086	0.004546	chr12:83792562-83792845	-0.2393	0.004562	284	TTS
Parp3	235587	-0.3549	0.000396	chr9:106474503-106474698	-0.2552	0.004459	196	intron
Per1	18626	-0.4836	0.036892	chr11:69096782-69097358	-0.2505	0.002781	577	Intergenic
Per1	18626	-0.4836	0.036892	chr11:69094677-69095463	-0.2480	0.002598	787	Intergenic
Per1	18626	-0.4836	0.036892	chr11:69098675-69099033	-0.2400	0.005018	359	promoter-TSS
Plekhg2	101497	-0.4135	0.000988	chr7:28375502-28375862	-0.2439	0.001153	361	Intergenic
Pllp	67801	-0.5750	0.000006	chr8:94703955-94704650	-0.2282	0.009023	696	Intergenic
Prex2	109294	-0.3745	0.023590	chr1:11144536-11144952	-0.1971	0.004511	417	intron
Prx	19153	-0.4932	0.000360	chr7:27507736-27508583	-0.2878	0.001827	848	intron
Relt	320100	-0.4846	0.047572	chr7:100862738-100863939	-0.2698	0.004297	848	promoter-TSS
Sema6b	20359	-0.5218	0.008366	chr17:56133848-56134242	-0.2566	0.005981	395	promoter-TSS
Septin9	53860	-0.2565	0.018349	chr11:117318422-117319018	-0.2687	0.004603	597	intron
Serpine2	20720	-0.2541	0.023590	chr1:80030816-80031601	-0.2201	0.008779	786	Intergenic
Sez6	20370	-0.2328	0.028089	chr11:77930748-77931223	-0.3063	0.000990	476	5' UTR
Slitrk1	76965	-0.3466	0.008437	chr14:108650093-108650367	-0.2667	0.000600	275	Intergenic
Tafa4	320701	0.2819	0.037206	chr6:96622700-96623311	0.2480	0.006358	612	intron

Supplemental Table 2 DARs annotated to myelin-related DEGs. 36 DARs annotated to DEGs related to myelin processes.
